# The durability of resin–dentine bonds are enhanced by epigallocatechin‐3‐gallate‐encapsulated nanohydroxyapatite/mesoporous silica

**DOI:** 10.1002/2211-5463.13521

**Published:** 2022-11-22

**Authors:** Taiyang Zhang, Wei Deng, Ying Zhang, Ming Liu, Yongchang Ling, Qiurong Sun

**Affiliations:** ^1^ Department of Stomatology The First Affiliated Hospital of Gannan Medical University Ganzhou China

**Keywords:** biomodification, epigallocatechin gallate, nanohydroxyapatite/mesoporous silica, resin–dentin bonds, thermal stability

## Abstract

Biomimetic nanohydroxyapatite (nHAp) has long been used as a biocompatible material for bone repair, bone regeneration, and bone reconstruction due to its low toxicity to local or systemic tissues. Various cross‐linkers have been employed to maintain the structure of collagen; these include epigallocatechin‐3‐gallate (EGCG), which can fortify the mechanical properties of collagen and withstand the degradation of collagenase. We hypothesized that EGCG combined with nHAp may promote resin–dentin bonding durability. Here, we examined the effect of epigallocatechin‐3‐gallate‐encapsulated nanohydroxyapatite/mesoporous silica (EGCG@nHAp@MSN) on thermal stability and remineralization capability of dentin collagen. Dentin slices (2 × 2 × 1 mm^3^) were obtained and completely demineralized in a 10% phosphoric acid water solution. The resulting dentin collagen matrix was incubated with deionized water, EGCG, nHAp@MSN, and EGCG@nHAp@MSN. The collagen thermal degradation temperature was assessed utilizing differential scanning calorimetry analysis, which indicated that EGCG, nHAp@MSN, and EGCG@nHAp@MSN reinforced collagen's capability to resist thermal degradation. EGCG@nHAp@MSN resulted in the highest increase in denaturation temperature. Thermogravimetric analysis showed that both nHAp@MSN and EGCG@nHAp@MSN achieved a higher residual mass than the EGCG and control groups. Fourier transform infrared spectroscopy was performed to examine the interaction between EGCG@nHAp@MSN and dentin collagen. The EGCG@nHAp@MSN sample exhibited stronger dentin microhardness and uppermost bond strength after thermocycling. EGCG significantly enhanced collagen's capability to resist thermal degradation. In summary, EGCG and nHAp@MSN may work together to assist the exposed collagen to improve resistance to thermal cycling and promote remineralization while also strengthening the durability of resin–dentin bonds.

## Graphical abstract

EGCG@nHAp@MSN acts as a reservoir of calcium and phosphate to facilitate crystal deposition and growth for dentin collagen remineralization. EGCG and nHAp can work together to assist the exposed collagen to improve resistance to thermal cycling and to achieve better mechanical characteristics, while also strengthening the durability of resin–dentine bonds.

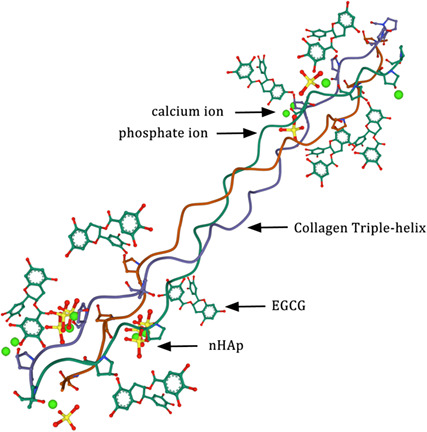

**Abbreviations**
DSC: differential scanning calorimetry analysis
EGCG: epigallocatechin‐3‐gallate
EGCG@nHAp@MSN: epigallocatechin‐3‐gallate‐encapsulated nanohydroxyapatite/mesoporous silica
FTIR‐ATR: Fourier transform infrared attenuated total reflectance
MSN: mesoporous silica nanoparticles
MTBS: microtensile bond strengths
nHAp: nanohydroxyapatite
TDT: thermal denaturation temperature
TGA: thermogravimetric analysis
VHN: Vickers hardness numbers
μTBS: microtensile bond strengths

The use of etch‐and‐rinse adhesives and self‐etching adhesives in dentin bonding has greatly improved the immediate resin–dentine bond strength in unique types of resin‐collagen hybrids [1]. The demineralized dentine collagen network acts as a scaffold for resin infiltration. One of the reasons underscoring the limited durability of resin–dentin bonds is the uncovered collagen fibers resulting from insufficient resin infiltration [2]. Researchers have attempted various methods to improve the physical and chemical properties of collagen fibrils. Remineralization of the demineralized collagen fibrils is one such solution [3, 4]. Biomimetic mineralized materials such as nanohydroxyapatite (nHAp) are of interest [5, 6]. nHAp has long been used as a biocompatible material for bone repair, bone regeneration, and bone reconstruction due to its low toxicity to local or systemic tissues. The continuous increases in Ca^2+^ and ${\mathrm{PO}}_{4}^{3-}$ concentration may lead to nHAp supersaturation and precipitation based on a stable collagen framework [7, 8, 9, 10, 11]. To maintain the structure of collagen and acquire better mechanical properties, various sorts of cross‐linkers have been adopted [12, 13, 14, 15]. One of the most potential cross‐linkers for collagen fibrils is epigallocatechin‐3‐gallate (EGCG) because it can fortify the mechanical properties of collagen and withstand the degradation of collagenase [16, 17]. Because of the favorable biological properties of nHAp, we hypothesized that EGCG combined with nHAp would promote resin–dentin bonding durability.

Mesoporous silica nanoparticles (MSN) have a large surface area to accommodate more nanoparticles as well as excellent adsorption performance [18, 19, 20]; they have been extensively applied in the biomedicine domain. MSN has an obvious advantage as a carrier for delivering functional nanoparticles, such as drugs and genes [21, 22, 23]. Epigallocatechin gallate‐encapsulated nanohydroxyapatite/mesoporous silica (EGCG@nHAp@MSN) was synthesized to block dentin tubules and reduce dentin hypersensitivity [24, 25]. The nHAp@EGCG‐modified collagen membranes could reinforce bone tissue regeneration in guided bone regeneration surgeries [17]. Thus, EGCG@nHAp@MSN is a prospective supplement for better resin–dentine bonds. Questions such as whether or not EGCG@nHAp@MSN can strengthen the durability of resin–dentin adhesion under artificial simulated pulpal pressure are still unclear. More effort is needed to determine the relationships between EGCG@nHAp@MSN pretreatment and the mechanical performance of demineralized dentin collagen.

The objective of this study was (a) to confirm the mechanical properties of demineralized collagen after preconditioning with EGCG, nHAp@MSN, and EGCG@nHAp@MSN; and (b) to assess resin–dentine bond durability after preconditioning. The hypotheses were that (a) EGCG@nHAp@MSN‐treated dentin collagen has better mechanical performance and can maintain the basic structure of the collagen backbone. (b) EGCG@nHAp@MSN‐treated dentin collagen can improve resin–dentine bond strength and bonding durability.

## Materials and methods

Intact human third molars were selected from 20‐ to 45‐year‐old anonymous subjects after obtaining the donors’ written informed consent according to a protocol approved by the ethical Committee of Gannan Medical University [no. LLSC2022082201]. This study was conducted in accordance with the Declaration of Helsinki and approved by the ethical Committee of Gannan Medical University. Dentin slices (2 × 2 × 1 mm^3^) were obtained. The volume and morphology of each sample were measured using a digital caliper accurate to the nearest 0.01 mm. All samples were put in a 10% phosphoric acid water solution for 18 h to produce a completely demineralized dentin collagen framework. The collagen was then rinsed in Milli‐Q water under agitation for 24 h [26]. FTIR‐ATR (Fourier transform infrared attenuated total reflectance; Bruker vertex 70, Karlsruhe, Germany) confirmed that there was no more mineral content in the dentin collagen, that is, the ${\mathrm{PO}}_{4}^{3-}$ peak at 1004 cm^−1^ disappeared. Dentin collagen specimens were then randomly assigned into three experimental groups and one blank control group for differential scanning calorimetry (DSC) analysis and thermogravimetric analysis (TGA). Eight teeth were obtained from eight donors, five dentin slabs (2 × 2 × 1 mm^3^) per tooth were obtained. Thus, 40 total specimens were used for DSC analysis and TGA. In microhardness measurements, 40 teeth were obtained from 40 donors, one dentin block (4 × 4 × 2.5 mm^3^) per tooth was obtained. Following mechanical testing, all dentin samples were analyzed using Fourier transform infrared spectroscopy.

Epigallocatechin‐3‐gallate (Sigma‐Aldrich, Saint Louis, MO, USA) was dissolved in deionized water to prepare 0.1% (w/v) EGCG solutions. The demineralized dentin collagen framework was then treated with 0.1% (w/v) EGCG at 25 °C for 1 h. The resulting EGCG‐modified dentin collagen framework was rinsed with deionized water three times and underwent freeze‐drying overnight. A control group was treated with deionized water under the same experimental circumstances. The nHAp@MSN compound was created through a homogeneous precipitation technique as described [18]. To synthesize the nHAp@MSN‐modified dentin collagen and the EGCG@nHAp@MSN‐modified dentin collagen, the control group and EGCG‐modified collagen specimens were submerged in 100 mg·mL^−1^ of nHAp@MSN solutions at 25 °C for 12 h. The specimens also went through freeze‐drying overnight [27]. The dentin collagen was treated with four groups: Group 1: control; Group 2: 0.1% EGCG; Group 3: 100 mg·mL^−1^ nHAp@MSN; and Group 4: 100 mg·mL^−1^ EGCG@nHAp@MSN.

### Differential scanning calorimetry analysis

The mechanical characteristics were analyzed by DSC measurements. Samples in each group (*n* = 40) were flushed with copious amounts of Milli‐Q water for 30 min. The thermal denaturation temperature (TDT) for each sample was measured by a differential scanning calorimeter (Netzsch‐Feinmahltechnik GmbH, Selb, Germany). All samples were lightly dried and placed in a DSC aluminum crucible. The dentin collagen framework individuals were constantly heated from 30 °C to 190 °C at a rate of 10 °C·min^−1^ under a nitrogen gas atmosphere. In the process, the initial and the maximum signal of TDT were detected and recorded [26].

### Thermogravimetric analysis

Samples in each group (*n* = 40) were sealed in an Al_2_O_3_ pan and connected to the TGA instruments (PERSEUS TG 209 F1 Libra; Netzsch, Bavaria, Germany). To stabilize the initial environment, the specimens were held at 20 °C for 5 min. They were then heated at 5 K·min^−1^ to 800 °C under a nitrogen atmosphere (20 mL·min^−1^) [28].

### Surface microhardness measurement

Another 40 teeth were obtained from 40 donors. One dentin block (4 × 4 × 2.5 mm^3^, with an intact dentin surface) per tooth was obtained. The selected sound teeth were cut and polished perpendicular to the long axis of the tooth to acquire coronal dentine slabs approximately 4 × 4 × 2.5 mm^3^ without enamel residual or destroying the pulp horn using a low‐speed diamond saw (Accotom‐50; Struers, Copenhagen, Denmark). Next, 40 dentine slabs were embedded in self‐cured resin with their dentine surfaces exposed for microhardness testing. All dentine blocks were demineralized using a pH cycling method to acquire a uniform demineralized layer [29]. The pH‐cycling model was used to simulate the dynamic changes in mineral content in natural caries process *in vitro*. Each sample was individually immersed in 5 mL of demineralizing solution (2.2 mm of CaCl_2_, 2.2 mm KH_2_PO_4_, 50 mm acetic acid, pH of 4.8) for 8 h, immersed in deionized water for 1 h and, subsequently, incubated in 5 mL of remineralizing solution (20 mm HEPES, 2.25 mm CaCl_2_, 1.35 mm KH_2_PO_4_, 130 mm KCl, pH of 7.0) for 15 h. The pH cycling procedure was carried out for 14 days, and all of the demineralizing and remineralizing solutions were made fresh and renewed daily [20, 30]. After 14 days, the demineralized dentin slabs were divided into four groups and immersed in deionized water, 0.1% EGCG solution, 100 mg·mL^−1^ of nHAp@MSN solution, and 100 mg·mL^−1^ of EGCG@nHAp@MSN for an hour.

The Vickers hardness numbers (VHN) of the demineralized dentin slabs were evaluated by a Vickers hardness tester (HXD‐1000TM; Taiming Optical Instrument Co., Shanghai, China). The load was 500 g for 10 s. Each sample was subjected to three different indentations spaced 100 mm from each other on the dentin surface. The mean surface microhardness of the three measurements from each sample was recorded as VHN in kgf·mm^−2^.

### Fourier transform infrared spectroscopy analysis on chemical properties

Following the mechanical test, all dentin samples were analyzed using Fourier transform infrared spectroscopy. The chemical characteristics of dentin collagen interacting with different reagents in each group were illustrated by an FTIR spectrophotometer (Fourier transform infrared attenuated total reflectance, Bruker vertex 70) and the spectra were acquired at an average of 32 scans from 400 to 4000 cm^−1^ at 25 °C [13].

### Microtensile bond strength evaluation

In terms of microtensile strength, 16 teeth were obtained from 16 donors. One dentin block (with the pulp exposed and an intact dentin surface) per tooth was obtained. Thus, 16 dentin blocks were used. The mid‐coronal flat surface of dentin was exposed parallel to the occlusal surface of the tooth using a low‐speed diamond saw with water cooling (Accotom‐50; Struers). Resin–dentine bonding was performed under simulated physiological pulpal pressure of 1.47 kPa. The results were obtained by employing an apparatus equipped with a water column 20 cm above the pulp chamber of the tooth [31]. The samples were randomly assigned into four groups. All the flat dentin surfaces were etched with 37% phosphoric acid (Heraeus Kulzer, Hanau, Germany) for 15 s, flushed with copious amounts of water for 10 s, and then lightly dried by water‐free air. The 16 dentin blocks were divided into four groups and treated with deionized water, 0.1% EGCG, 100 mg·mL^−1^ of nHAp@MSN, or 100 mg·mL^−1^ of EGCG@nHAp@MSN with agitation for 120 s. Every group had four dentin blocks.

Next, Adper Single Bond 2 was applied to the dentin for 15 s according to the instructions. The resin composite was performed on dentin in two layers—2 mm each followed by light curing for 20 s (Elipar 2500 Halogen Curing Light; Minnesota Mining and Manufacturing, Saint Paul, MN, USA). Every group contained four resin–dentine specimens. The bonded test specimens from each group were subsequently assigned into two subgroups: The first subgroup was incubated in deionized water at 37 °C for 24 h; the second subgroup went through 5000 thermal cycles in water (between 5 °C and 55 °C, with a dwell time of 30 s). Thereafter, all the resin–dentine composites were cut into resin–dentin beams of 0.9 × 0.9 mm^2^ in dimension. Five resin–dentine beams could be obtained per resin–dentine bonding specimen. Ten resin–dentine beams from the first subgroup stand for the immediate μTBS. Ten resin–dentine beams from the second subgroup stand for the μTBS after thermal cycling. Beams with enamel residual or situated peripherally were excluded. The remaining qualified beams were employed for μTBS testing. The resin–dentin beams were stuck to a testing jig using cyanoacrylate adhesive, and a tensile force was performed with a universal testing machine (T‐6102K; Bisco, Schaumburg, IL, USA) at a crosshead speed of 1 mm·min^−1^ until failure. Beams that failed prematurely were excluded from the statistical analysis [32].

### Statistical analysis

The statistical analysis was processed via spss 19.0 (IBM Corporation, Armonk, NY, USA) with a significance level of *P* < 0.05. All results were analyzed as mean value ± SD deviation. Differences between groups were conducted utilizing one‐way analysis of variance followed by Tukey's multiple comparison tests.

## Results

### Differential scanning calorimetry analysis

**Fig. 1:**
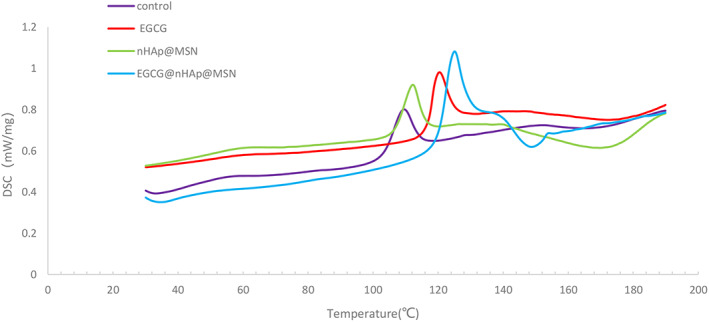
Differential scanning calorimetry analysis of completely demineralized dentin collagen treated with deionized water, EGCG, nHAp@MSN, and EGCG@nHAp@MSN.

The DSC analysis showed that EGCG, nHAp@MSN, and EGCG@nHAp@MSN preconditioning increased the initial denaturation temperature of the dentin collagen relative to the deionized water group (Fig. 1). The three preconditioners significantly increased the signal max temperature. The deionized water group presented a control initial denaturation temperature of 99.99 ± 1.20 °C and a maximum TDT of 109.40 ± 2.48 °C (Table 1). The initial thermal denaturation and signal max temperature of dentin collagen preconditioned with EGCG@nHAp@MSN was 118.00 ± 2.73 °C and 126.00 ± 1.88 °C. The TDT of the EGCG@nHAp@MSN preconditioning group increased the most of all groups. There was a significant difference between the 0.1% EGCG group and 100 mg·mL^−1^ of nHAp@MSN group (Table 1).

**Table 1:** Changes in thermal degradation temperature in completely demineralized dentin matrix after pretreatment with deionized water, EGCG, nHAp@MSN, and EGCG@nHAp@MSN. Values are mean ± SD in °C, *n* = 10. Different superscript numbers in a row indicate significant differences (*P* < 0.05).

Dentin treatment	Onset temperature (°C)	Signal max temperature (°C)
Deionized water	99.99 ± 1.20^A^	109.40 ± 2.48^a^
0.1% EGCG	113.48 ± 2.03^C^	120.23 ± 1.67^c^
100 mg·mL^−1^ nHAp@MSN	104.54 ± 2.55^B^	112.84 ± 2.69^b^
100 mg·mL^−1^ EGCG@nHAp@MSN	118.00 ± 2.73^D^	126.00 ± 1.88^d^

### Thermogravimetric analysis

**Fig. 2:**
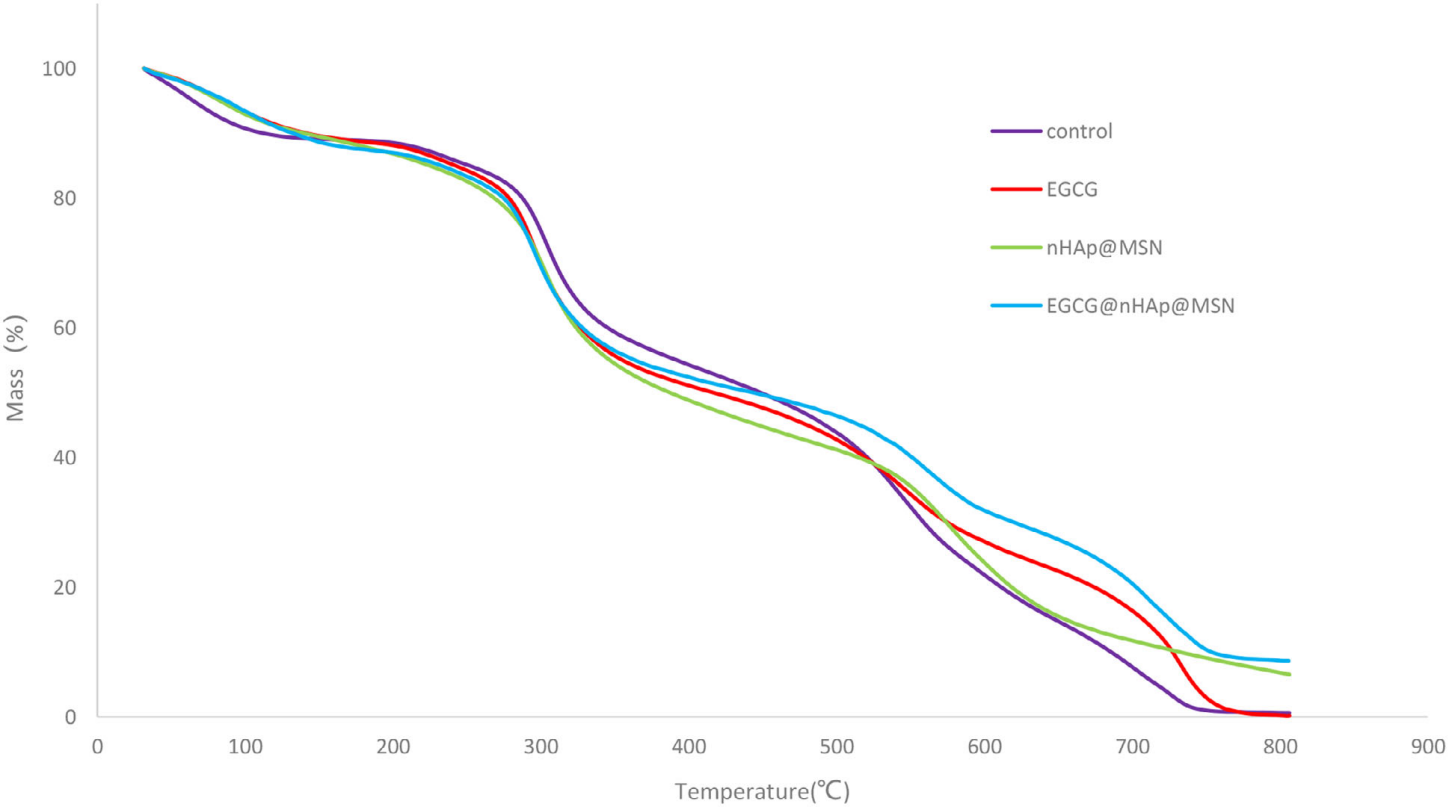
Thermogravimetric analysis of demineralized dentin collagen matrices treated with deionized water, EGCG, nHAp@MSN, and EGCG@nHAp@MSN.

The thermogravimetric analysis demonstrated that dentin collagens pretreated with nHAp@MSN or EGCG@nHAp@MSN presented a larger portion of residual mass (Fig. 2). The residual mass of dentin collagens in nHAp@MSN and EGCG@nHAp@MSN groups were 6.50 ± 0.61% and 8.62 ± 1.19%, respectively. The results showed a significant difference between the nHAp@MSN and EGCG@nHAp@MSN groups. The residual mass of dentin collagens in deionized water or 0.1% EGCG group were 0.52 ± 0.18% and 0.59 ± 0.12% respectively. There is no significant difference between the deionized group and the EGCG group (Table 2).

**Table 2:** Changes of residual mass in completely demineralized dentin matrix after pretreatment with deionized water, EGCG, nHAp@MSN, and EGCG@nHAp@MSN. Values are mean ± SD in %, *n* = 10. Different superscript numbers in a row indicate significant differences (*P* < 0.05).

Dentin treatment	Residual mass (%)
Deionized water	0.52 ± 0.18^A^
0.1% EGCG	0.59 ± 0.12^A^
100 mg·mL^−1^ nHAp@MSN	6.50 ± 0.61^B^
100 mg·mL^−1^ EGCG@nHAp@MSN	8.62 ± 1.19^C^

### Surface microhardness measurements

The results in Table 3 show the surface microhardness measurements of the dentin surface. The nHAp@MSN and EGCG@nHAp@MSN groups displayed a higher VHN than the control group and the 0.1% EGCG group. The results showed no significant difference between the control group and the 0.1% EGCG group. Among the four groups, the EGCG@nHAp@MSN group presented the highest VHN after remineralization due to the dual function of EGCG and nHAp@MSN.

**Table 3:** Effect of different pretreatments on dentine surface microhardness. Values are mean ± SD in MPa, *n* = 10. Different superscript numbers in a row indicate significant differences (*P* < 0.05).

Dentin treatment	VHN
Deionized water	38.51 ± 2.37^A^
0.1% EGCG	39.05 ± 2.38^A^
100 mg·mL^−1^ nHAp@MSN	43.44 ± 2.22^B^
100 mg·mL^−1^ EGCG@nHAp@MSN	46.44 ± 1.86^C^

### Fourier transform infrared spectroscopy

**Fig. 3:**
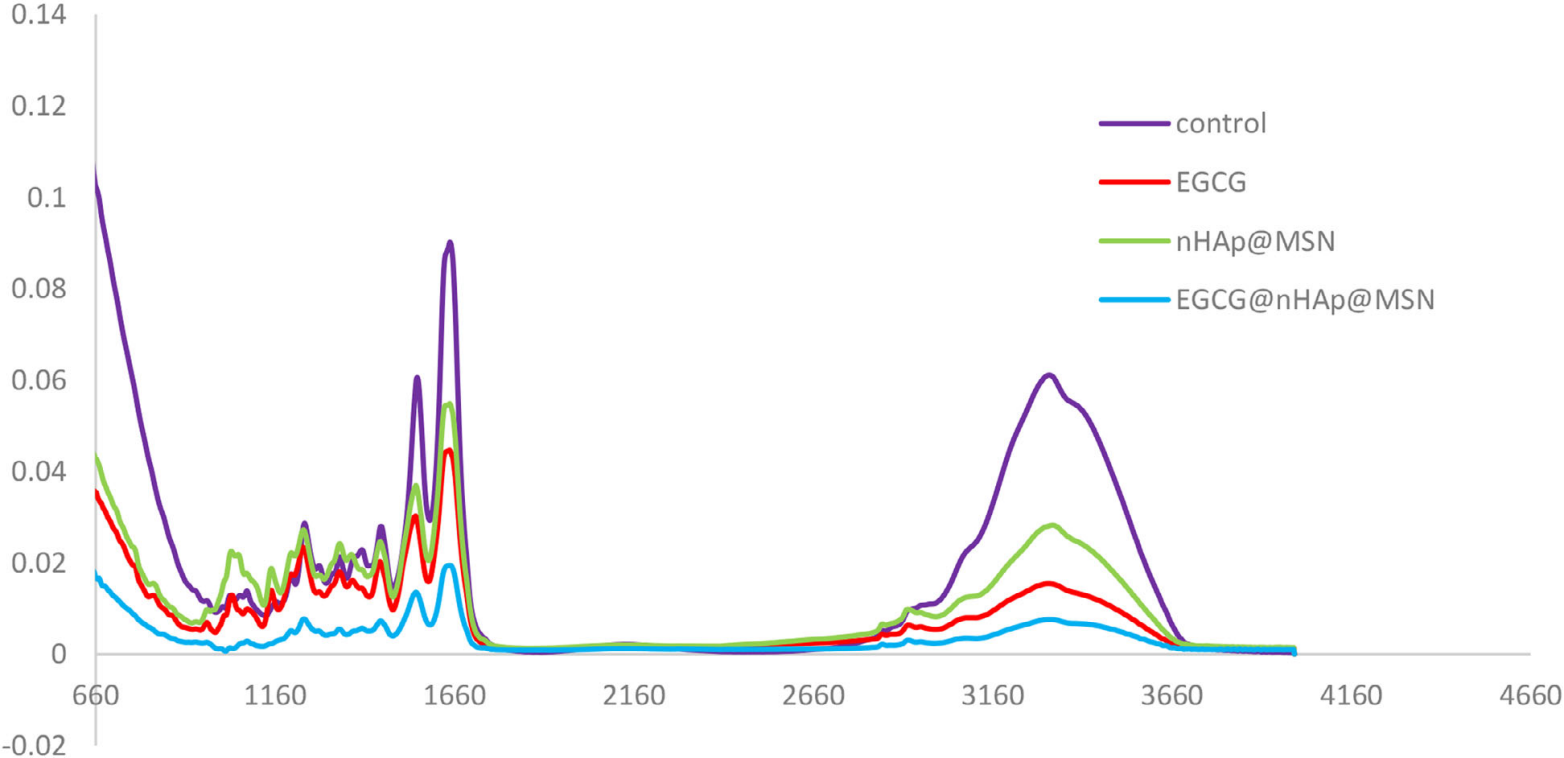
Fourier transform infrared spectroscopy of demineralized dentin collagen matrices treated with deionized water, EGCG, nHAp@MSN, and EGCG@nHAp@MSN.

Fourier transform infrared spectroscopy analyzed the chemical constituents of dentin collagen (Fig. 3). The infrared spectra showed five typical absorption peaks in the control group: amide A (3321 cm^−1^), amide B (3062 cm^−1^), amide I (1650 cm^−1^), amide II (1552 cm^−1^), and amide III (1242 cm^−1^). The strength of the typical absorption peaks of the amide was significantly reduced in the other three groups. The EGCG@nHAp@MSN group as well as the amide II and III bands were close to disappearing, and the amide III band red‐shifted. The amide A, I, and II bands are widened and weakened in the three experimental groups, thus indicating interactions between the EGCG, nHAp@MSN, and collagen.

### MTBS evaluation

Table 4 showed the microtensile bond strengths of resin–dentin bonds in each group. EGCG, nHAp@MSN, and EGCG@nHAp@MSN showed higher immediate microtensile bond strengths than the control group. The bonding strength decreased obviously in each of the four groups after thermocycling.

**Table 4:** MTBS of resin–dentin bonds with EGCG, nHAp@MSN, and EGCG@nHAp@MSN preconditioner under simulated pulp pressure. Values are mean ± SD in MPa, *n* = 10 beams. Different numerical superscripts in one row represent the significant difference (*P* < 0.05); different letter superscripts in one column represent significant differences (*P* < 0.05).

Group	Noncycled (MPa)	Thermocycled (MPa)	% Change from thermocycling
Deionized water	14.49 ± 1.70^A,1^	7.20 ± 1.10^a,2^	−50.31
0.1% EGCG	17.36 ± 1.34^B,1^	11.52 ± 1.89^b,2^	−33.64
100 mg·mL^−1^ nHAp@MSN	17.57 ± 2.06^B,1^	10.55 ± 2.55^b,2^	−39.95
100 mg·mL^−1^ EGCG@nHAp@MSN	18.74 ± 2.65^B,1^	14.41 ± 1.87^c,2^	−23.11

The EGCG@nHAp@MSN pretreated group had no significance in the immediate microtensile bond strength versus the nHAp@MSN and EGCG@nHAp@MSN groups, but there was a higher bond strength after thermocycling. The percent change after thermocycling in each group was 50.31%, 33.64%, 39.95%, and 23.11%. After thermocycling, the EGCG@nHAp@MSN group had the highest bond strength among the four groups.

## Discussion

This paper studies the impact of EGCG@nHAp@MSN on the thermal resistance of dentin collagen and the microhardness of demineralized dentin substrate. The results show that EGCG@nHAp@MSN achieved better mechanical properties than EGCG or nHAp@MSN. We also used FTIR to clarify the interaction between experimental agents and dentin collagen. The second variable that determines the microtensile bonding strength is thermal cycling. Results show that the application of EGCG@nHAp@MSN on demineralized dentin collagen has a positive effect on resin–dentine bond durability.

Differential scanning calorimetry was used to measure the TDT of collagen materials. Collagen denaturation implies that a triple helix structure of collagen molecules turns into a random coil structure due to the increased temperature. The denaturation temperature of collagen refers to the temperature when the unique triple helix structure of collagen is completely destroyed. The chemical crosslinking method can increase the noncovalent and covalent crosslinking points in the collagen material to limit the movement of the collagen chain segment, stabilize the collagen triple helix structure, and increase the denaturation temperature of collagen. In this study, the EGCG‐crosslinked collagen matrices had increased internal hydrogen bond and covalent bond binding, which restricts the peptide chain activity in collagen molecules and increases the difficulty of the phase transition [33]. Therefore, the heat energy and temperature required for collagen denaturation increase correspondingly, that is, the thermal stability of collagen materials is enhanced. More complex molecules such as polyphenol crosslinkers have more groups that react with collagen and in turn, have a higher degree of crosslinking—this, in turn, leads to higher thermal stability after crosslinking. The deposition of nHAp in collagen matrices also improved the thermal ability of collagen. The dual function of EGCG and nHAp achieved the highest TDT in the EGCG@nHAp@MSN group.

Thermogravimetric analysis showed that the residual mass of the dentin collagen in the control and EGCG groups is close to zero. The residual mass is about 6.52 ± 1.78% in the nHAp@MSN group and about 8.61 ± 1.25% in the EGCG@nHAp@MSN group. The residual mass in the EGCG@nHAp@MSN group was significant versus the nHAp@MSN group suggesting that the EGCG@nHAp@MSN group had better remineralization than the nHAp@MSN group. Stabilization of the organic matrix network may support mineral deposition and prevent mineral loss. The dentin demineralization rate decreases when the amount of stable collagen increases. Previous studies suggest that polyphenol has a synergistic effect on demineralized dentin collagen when combined with other remineralizing agents [34, 35]. There is no sufficient evidence indicating the existence of intrafibrillar mineralization, but polyphenol with a remineralizing agent seems able to strengthen the collagen framework and promote extrafibrillar collagen fibril remineralization [30].

The nHAp provides a source of calcium and phosphate ions and can realize extrafibrillar remineralization. Prior studies indicated that the nHAp can act as a reservoir on demineralized tooth surfaces by releasing a continuous amount of calcium and phosphate ions to facilitate crystal growth and deposition [6]. nHAp does not have a considerable collagen‐protective effect leading to inhibited collagen breakdown and a stabilized collagen matrix. However, EGCG only has a weak remineralization capability but has multiple beneficial effects on dentin collagen [12]. It is meaningful to combine EGCG with nHAp for cross‐linking of collagen and remineralization of demineralized dentin. The mesoporous silica act as a carrier to transport sustainable supplies of calcium, phosphate ions, and EGCG molecules to the demineralized dentin collagen matrix, which can promote the biomodification of demineralized collagen fibrils and in turn protect the exposed collagen fibrils from the degradation of collagenolytic enzymes and accelerate extrafibrillar remineralization.

Nanomechanical testing is essential for deeper insight into the recovery of mechanical strength at the nanostructural level [20]. Microhardness measurements are one of the methods used to assess the nanomechanical properties. There are also other nanomechanical tests, such as three‐point bending and elastic modulus. Our goal here was to understand whether remineralization of collagen improved the microhardness of dentin. The EGCG@nHAp@MSN group presented the highest VHN after remineralization. EGCG positively affects the remineralization ability of nHAp in artificial dentin caries and may be a potential crosslinking agent for remineralization. A previous study showed that polyphenol chelates with Ca^2+^, which reinforces mineral deposition on the dentin surface. Polyphenol is also acidic, and an acidic and nonoxidizing environment can promote collagen crosslinking and mineral deposition [36].

Fourier transform infrared spectroscopy shows interactions between preconditioners and collagen, The demineralized dentin collagen in the control group has five typical absorption peaks: 3321 cm^−1^ is the amide A band peak (representing –OH and NH telescopic vibration), 3062 cm^−1^ is the amide B band peak (representing = C–H and –NH^3+^ telescopic vibration), 1600–1700 cm^−1^ is the amide I band peak (representing C=O telescopic vibration and NH bending vibration), 1550–1600 cm^−1^ is the amide II band peak (representing N–H bending vibration and C–N telescopic vibration), and 1200–1360 cm^−1^ is the amide III band peak (representing N–H bending vibration and C–N telescopic vibration) [37]. Compared to unmodified collagen, the position of these major amide bands in modified collagen is almost maintained in the original position after crosslinking—the amide I band associated with the collagen triple helix is almost maintained in the same position. This suggests that the collagen triple helix in the dentin collagen is retained after the introduction of polyphenol crosslinkers or nHAp@MSN, and the natural structure of collagen is not destroyed. The position and strength of collagen characteristic peaks indicate polyphenol crosslinkers, nHAp@MSN can interact with collagen molecules.

In the EGCG group, amide A, B, I, and II bands are weakened and widened, thus indicating a hydrogen bond and covalent bond interaction between the polyphenol crosslinker and collagen. In addition, a greater number of functional groups reacting with collagen in the polyphenol crosslinker implies a stronger interaction effect with collagen and a weaker absorption peak strength. In the nHAp@MSN group, the carboxyl and carbonyl groups on the collagen are the two nucleation sites of HAp biomineralization. The oxygen atoms on the carboxyl and carbonyl groups are coordinated with Ca^2+^ in solution to become the core of homogeneous nucleation. The carbonyl groups and carboxyl groups are all or partially wrapped by the inorganic minerals, thus leading to a decrease in the intensity of the infrared absorption peaks of the amide A, B, I, and II bands as well as redshifts in the amide III band. A higher degree of mineralization leads to more bound carboxyl groups and in turn smaller peaks for amide A and B bands as well as amide I and II bands.

The EGCG@nHAp@MSN group has the weakest and widest amide A, B, I, II, and III bands. The amide II and III bands were close to disappearing. The amide III band redshifts. The characteristic peaks of collagen in some regions after crosslinking are transferred to a lower wavenumber, which also indicates that the interaction between collagen and EGCG@nHAp@MSN is much stronger than in the other three groups. The formation of hydrogen bonds reduces the bond force constant K; the absorption peak shifts toward the low wave number and the higher offset indicates a higher degree of hydrogen bond binding. EGCG stabilized the dentin collagen and promoted Ca^2+^ and ${\mathrm{PO}}_{4}^{3-}$ deposition. Instead of shrinkage, EGCG preconditioning may increase the diameters of fibers, which may also contribute to the entry and distribution of Ca^2+^ and ${\mathrm{PO}}_{4}^{3-}$. These two agents reinforce each other, not constrain each other [27].

We did not see the ${\mathrm{PO}}_{4}^{3-}$ band in the control group or EGCG group. We saw a typical band around 1040 cm^−1^ for ${\mathrm{PO}}_{4}^{3-}$ vibration in the nHAp@MSN group. The ${\mathrm{PO}}_{4}^{3-}$ band nearly disappeared in the EGCG@nHAp@MSN group. The presence of this phenomenon may be due to the following reasons. First, we can see that the binding ability of EGCG to collagen is stronger than that of nHAp to collagen. When EGCG and nHAP competitively bind the carbonyl and carboxyl groups on collagen, the presence of EGCG may restrict the movement of phosphate ions due to space limitations. This weakens the vibration peak of phosphate ions. Second, during the process of remineralization, HAp precipitation may form highly crystalline, as well as poorly crystalline, apatite. Poorly crystalline apatite tends to be reactive and highly soluble, and may dissolve in the weak acid situation provided by EGCG solution. Only the highly crystalline apatite binding the collagen tightly can remain in an EGCG environment. The vibration of ${\mathrm{PO}}_{4}^{3-}$ may be restricted in this situation, so X‐ray diffraction (XRD) is needed to analyze the amount of mineral.

The addition of nHAp@MSN did not reduce the immediate microtensile bond strength between acid‐etching adhesives and dentin. There is no significant difference among the EGCG group, nHAp@MSN group, and EGCG@nHAp@MSN in the immediate microtensile bond strength. After thermocycling, the EGCG@nHAp@MSN group had the highest bond strength versus the other three groups due to the acid‐resistant stability and remineralization potential. EGCG@nHAp@MSN‐modified dentin collagen not only maintained the basic structure of the collagen backbone but also presented stronger mechanical characteristics.

In the microtensile bond strength test, Adper Single Bond 2, a type of etch‐and‐rinse adhesive that does not contain acid monomer, was adopted to form a resin–dentin specimen. If we chose a self‐etching adhesive with acid monomer, then the acid monomer might affect the remineralization function of nHAp or it might dissolve the inorganic minerals that had just formed. Further study is needed. In clinical practice, the occurrence of nano‐leakage at the bonding interface is closely related to the acidic environment produced by bacteria and various matrix‐metalloproteinases. Therefore, the acid‐resistant stability is also a crucial standard to evaluate the ability of EGCG@nHAp@MSN. Future research will add more influencing factors to make the research environment closer to the clinical situation.

## Conclusion

This study suggests that EGCG significantly reinforced collagen's resistance to thermal degradation. Furthermore, EGCG and nHAp@MSN can work together to acquire better remineralization of dentin collagen and promote resin–dentin bond durability. EGCG@nHAp@MSN‐modified dentin collagen achieved better mechanical characteristics without destroying the collagen backbone.

## Conflict of interest

The authors declare no conflict of interest.

## Author contributions

QS conceived and supervised the study; WD designed experiments; TZ and YZ performed experiments and analyzed data; QS and ML wrote the manuscript; and YL made manuscript revisions; All authors read and approved the final manuscript.

## Data Availability

The data that support the findings of the present study are available from the corresponding author upon reasonable request.
